# The Influence of Caramel Carbon Quantum Dots and Caramel on Platelet Aggregation, Protein Glycation and Lipid Peroxidation

**DOI:** 10.3390/antiox13010013

**Published:** 2023-12-20

**Authors:** Magdalena Kotańska, Konrad Wojtaszek, Monika Kubacka, Marek Bednarski, Noemi Nicosia, Marek Wojnicki

**Affiliations:** 1 Department of Pharmacological Screening, Jagiellonian University Medical College, 9 Medyczna St., 30-688 Krakow, Poland; marek.bednarski@uj.edu.pl; 2 Faculty of Non-Ferrous Metals, AGH University of Krakow, Mickiewicza Ave., 30-059 Krakow, Poland; kwojtasz@agh.edu.pl; 3 Department of Pharmacodynamics, Jagiellonian University Medical College, 9 Medyczna St., 30-688 Krakow, Poland; monika.kubacka@uj.edu.pl; 4 PhD Program in Neuroscience, Department of Medicine and Surgery, University of Milano-Bicocca, 20126 Milan, Italy

**Keywords:** caramel, caramel carbon quantum dots, caramelization, platelet aggregation, protein glycation, lipid peroxidation, erythritol, xylitol

## Abstract

Caramel, defined as a coloring agent and as an antioxidant, is used in several kinds of food products and is consumed by many people in different amounts. In our research we showed that the caramelization of sucrose under special conditions leads to the formation of carbon quantum dots (CQDs). So, it makes sense that humans also consume this type of CQDs, and it is theoretically possible for these particles to affect the body. Despite an increasing number of studies describing different types of CQDs, their biosafety is still not clearly understood. In our in vitro research, we examined the effects on platelet aggregation, protein glycation and lipid peroxidation of CQDs and caramel formed from a 20% sucrose solution. In vitro aggregation tests were conducted using freshly collected whole rat blood in a multiplate platelet function analyzer and measurer of electric impedance. The cytotoxic effect of the tested solutions on blood platelets was evaluated based on the release of lactate dehydrogenase. The formation of glycated bovine serum albumin was measured as fluorescence intensity and fructosamine level. The reducing power of the solutions was determined in adipose tissue, and their effect on lipid peroxidation in adipose tissue in vitro was also assessed. By measuring the intensity of hemolysis after incubation in solutions with red blood cell, we assessed their influence on the integration of the red blood cell membrane. All tests were performed in comparison with glucose and fructose and other frequently used sweeteners, such as erythritol and xylitol. Our study showed that caramel and CQDs formed from caramel may influence the glycation process and integrity of the red blood cell membrane, but unlike glucose and fructose, they decrease lipid peroxidation and may reduce Fe (III). Additionally, it is unlikely that they affect platelet aggregation. Compared to glucose and fructose, they may be safer for patients with metabolic disorders; however, further research is needed on the safety and biological activity of caramel and CQD.

## 1. Introduction

In recent years, global sugar overconsumption has raised concerns about its serious impact on public health. Evidence supports a solid correlation between the excessive intake of refined sugar and a higher incidence of ailments such as dental decay, type II diabetes, obesity, cardiovascular disorders, and other non-communicable diseases. Ongoing studies in the field of nutrition and health sciences highlight the potential use of sugar substitutes in weight and obesity management [1]. Over the past few decades, food and pharmaceutical companies have progressively replaced sucrose with artificial sweeteners [2].

Sweeteners are classified based on their origin, nutritional value, texture, and technical function. Natural sugars include glucose, fructose, and sucrose, and natural sweeteners include sugar alcohols (or polyols). Natural compounds include Stevia glycosides [3].

Sugars are subject to various thermal treatment processes, the most common of which being the caramelization process. The controlled heat treatment of carbohydrates produces caramel. The carbohydrate raw materials used are the monomers glucose and fructose, or their polymers (such as glucose syrups, sucrose or invert sugars, and dextrose) [4]. Caramel is polymeric in its character. Among the numerous products of carbohydrate caramelization, the volatile substances, and the group of non-volatile carbohydrate oligocondensation products, are the most thoroughly characterized. As is well known, caramel is produced for its aroma and taste properties. Volatile compounds such as acetylfuran, furfurol, 5-hydroxymethylfurfural, 3-hydroxy- 2-acetylfuran and others are responsible for the aroma of caramel. Among the non-volatile compounds that make up caramel are caramelan, caramelen, and caramelin. These compounds are assumed to be polymeric in nature, with chain lengths of C24, C56, and C96, respectively [4]. Caramel has been used in foods and beverages for over 150 years and is globally regulated as a color additive [5]. Caramel color, ranging from the palest yellow to the deepest brown, accounts for more than 80% (by weight) of all colorants added to the foods humans eat and drink [4]. Available studies indicate that the intake of caramel colors and constituents does not pose any undue safety risks [5].

The caramelization of sugars under special conditions leads to the formation of carbon quantum dots (CQDs). In recent years, great attention has been paid to carbon quantum dots created from various substrates and their properties in terms of safety or beneficial effects on the body. CQDs are a type of novel, small nanomaterial with photoluminescent behaviors [6]. Carbon quantum dots are a kind of scientific novelty, and they were discovered in 2006 [7]. The last 10 years have seen intensive research into their synthesis [8], and the last 8 years into their bio-chemical properties. For obvious reasons, i.e., the lack of content of toxic primes such as Cd, Se, As, Pb, etc., carbon quantum dots are seen as a candidate for direct human contact applications [9]. They are being processed as a drug carrier, disease marker, etc. However, the amount of research on their safety is very small. It has been assumed that since they are made from non-toxic ingredients, they themselves are also non-toxic [10]. However, it should be noted and emphasized that nanomaterials and, in particular, quantum dots, have the very feature of interest to physicists and chemists, namely, that their properties change with size. Thus, the arbitrary assumption that they are non-toxic because they are composed of non-toxic components is incorrect. Despite an increasing number of studies describing different types of fluorescent carbon quantum dots [11], their biosafety is still not clearly understood. Therefore, within the framework of this work, we took advantage of the fact that caramel, as a sweetener formed from sucrose, is also used for the synthesis of CQDs [12]. Moreover, caramel itself exhibits photoluminescence, which is probably related to the formation of small amounts of CQDs during the process. Thus, it seems obvious that the two materials should be analyzed separately, bearing in mind, however, that they share common roots.

The sugar alcohols xylitol and erythritol have become a major target of interest due to their numerous health benefits, including anti-hyperglycaemic, anti-diabetic, and anti-obesogenic effects [13]. While xylitol’s sweetness is comparable to that of sucrose, erythritol is about 30% less sweet. Their lower caloric values, minimal insulinemic response, and low glycaemic index makes them preferred sweeteners for diabetics [14]. It is considered that sugar alcohols might mitigate diabetes-associated oxidative stress due to valuable antioxidant properties, which have been reported in recent studies [13,14,15]. By influencing glucose metabolism and reducing lipid peroxidation, erythritol and xylitol can potentially counteract the oxidative stress implicated in diabetes pathogenesis [13]. Moreover, xylitol was found to be more effective in reducing the accumulation of visceral fat in the liver and in improving diabetic parameters such as blood glucose, serum fructosamine and glucose tolerance compared to erythritol [15]. Although sugar alcohols are universally classified as safe, a recent study has found a correlation between erythritol consumption and increased platelet aggregation, along with thrombus formation, in human blood [2]. Additionally, there is growing epidemiological evidence linking the consumption of artificial sweeteners to adverse cardiometabolic phenotypes, such as weight gain [16], insulin resistance [17,18], cardiovascular disease [19], and cardiovascular mortality [20,21].

We have not found any data in the literature regarding the impact of caramel, derived from sucrose, and CQDs on the processes of platelet aggregation, protein glycation and lipid peroxidation. Therefore, we decided to perform comparative tests to determine the effects of simple sugars such as glucose and fructose, of sweeteners such as erythritol and xylitol, and of caramel and carbon quantum dots on these processes. We also determined CQDs’ reducing properties in in vitro tests.

## 2. Experimental Methods

### 2.1. Chemicals and Reagents

Bovine serum albumin (BSA), Tris, HEPES-NaOH, disodium edetate, theophylline, TEP, trichloroacetic acid (TCA), 2-thiobarbituric acid (TBA), 2,4,6-tris(2-pyridyl)-s-triazine (TPTZ), glucose, fructose and 0.1 M phosphate buffer (PBS) were purchased from Sigma-Aldrich Chemie GmbH (Taufkirchen, Germany). 1-deoxy-1-morpholino-fructose (1-DMF) and nitroblue-tetrazolium (NBT) were purchased from BLD Pharmatech GmbH (Kaiserslautern, Germany). Erythritol was purchased from SHG GROUP (Pustynia, Poland). Xylitol was purchased from Nanga (Złotów, Poland) and collagen was from Hyphen Biomed (Neuville-sur-Oise, France). KCl, Na_2_CO_3_, NaCl, CaCl_2_, KH_2_PO_4_, MgCl × 6 H_2_O, NaHCO_3_, FeCl_3_ × 6 H_2_O, FeSO_4_ × 7 H_2_, sodium citrate and citric acid were purchased from Avantor Performance Materials Poland S.A. (Gliwice, Poland).

### 2.2. Preparation of Caramel and Carbon Quantum Dots (CQDs)

The caramel was obtained as follows: 20 g of sucrose were dissolved in 100 mL of deionized water; 25 mL of this solution was placed in a conical flask and positioned on a heating plate with a thermometer. The caramelization process was carried out at a temperature of 160 °C for 2 h and 15 min, with free access to air. After cooling, the solution was filtered through filter paper, and the filtrate was transferred to a 50 mL volumetric flask and diluted with deionized water.

Carbon quantum dots (CQDs) were prepared by modifying a method adopted from the literature [22]. To achieve this, 25 mL of a 20 g/100 mL sucrose solution was placed in a Teflon-lined pressure reactor. Subsequently, the reactor was placed in an oven at a temperature of 160 °C for a duration of 2 h and 15 min. The reactor was then cooled along with the oven. After cooling, the solution was filtered through filter paper, and the filtrate was transferred to a 50 mL volumetric flask and diluted with deionized water. A black powdery mass resembling activated charcoal was filtered off. The UV-Vis spectra of sucrose, caramel, and quantum dots were investigated using a Shimadzu spectrophotometer. Using the Dynamic Light Scattering method (DLS), the sizes of carbon quantum dots were determined, and their zeta potential was calculated.

To determine the doses used in in vitro studies, specific volumes of suspensions containing caramel and CQDs were evaporated at a temperature of 100 °C. Based on this, the dry mass content was determined.

It was determined that the concentration of CQDs in the base solution was 0.0116 g/mL, while in the case of caramel, it was 0.0971 g/mL.

### 2.3. Tested Solutions

The concentrations of the tested solutions were selected based on the results in the literature [23,24,25,26,27,28]. The following solutions were tested:

1. Dilutions of the base CQD solution—three-fold (Q1), ten-fold (Q2), thirty-fold (Q3) and one-hundred-fold (Q4);
2. Dilutions of the base caramel solution—three-fold (C1), ten-fold (C2), thirty-fold (C3) and one-hundred-fold (C4);
3. 500 mM sucrose (S500);
4. Glucose—500 mM (G 500), 50 mM (G 50);
5. Fructose—500 mM (F 500), 50 mM (F50);
6. 250 mM glucose + 250 mM fructose (G + F);
7. Erythritol—500 mM (E 500), 50 mM (E 50), 5 mM (E 5);
8. Xylitol—500 mM (X 500), 50 mM (X 50), 5 mM (X 5).

### 2.4. Whole Blood Aggregation

In vitro aggregation tests were conducted using freshly collected whole rat blood in a multiplate platelet function analyzer (Roche Diagnostic, Mannheim, Germany), with the five-channel aggregometer based on measurements of electric impedance, as detailed in previous procedures [29]. Upon activation, platelets adhere to and progressively aggregate on a duplicate metal sensor in the analyzer test cuvette. This leads to a change in resistance, which is proportional to the number of platelets adhering to the electrodes.

Blood was drawn from the carotid of rats into Hirudin blood tubes (S-Monovette, Hirudin, Sarstedt, Germany). Then, 300 μL of Hirudin anticoagulated blood was mixed with 300 μL of prewarmed isotonic saline solution containing tested substance or vehicle (saline) and preincubated for 3 min at 37 °C with continuous stirring. Aggregation was induced by adding collagen (0.96 µL of 1 mg/mL solution, final concentration 1.6 µg/mL). Activated platelet function was recorded for 6 min. The multiplate software (version V2.05) analyzed the area under the curve (AUC) of the clotting process of each measurement and calculated the mean values.

### 2.5. Impact on Platelet Viability (Cytotoxicity Test)

The platelet viability of freshly collected platelet-rich rat plasma was determined according Kubacka et al., 2023 [30]. Whole blood (about 5 mL) was collected from rats in a glass tube containing 0.5 mL of PECT medium (94 nM prostaglandin E1, 0.63 mM Na_2_CO_3_, 90 mM disodium edetate, and 10 mM theophylline). A density barrier was created by combining 5 mL of 1.320 g/mL 60% iodixanol stock solution (OptiPrep density gradient medium, Sigma-Aldrich, Sant Louis, MO, USA) with 22 mL of diluent (0.85% NaCl, 20 mM HEPES-NaOH, pH 7.4, 1 mM disodium edetate). For platelet separation, 3 mL of each sample was layered over 5 mL of the 1.063 g/mL density barrier. Samples were centrifuged at 350× *g* for 15 min at 20 °C [31]. The platelets were then suspended in Barber’s buffer (0.14 M NaCl, 0.014 M Tris, 10 mM glucose; pH 7.4). The dilutions were 10× or 20×.

The cytotoxic effect of the tested solutions on blood platelets was evaluated based on the release of lactate dehydrogenase (LDH), according to the instructions given by the kit manufacturer (Cytotoxicity Detection KitPLUS, Cat. No. 04744926001, Merck, Darmstadt, Germany). The time of platelet incubation of the tested solutions was 10 min.

Absorbance is an additive quantity. Therefore, a correction of the results was introduced by subtracting the absorbance of CQDs and/or caramel from the measured values.

### 2.6. In Vitro Protein Glycation

#### 2.6.1. Preparation of Glycated BSA Samples

The formation of glycated BSA was determined according to the modified method of Vinson and Howard, 1996 [32]. In brief, BSA (10 mg/mL in 100 mM sodium phosphate buffer, pH 7.4) was incubated with the tested solutions in 100 mM sodium phosphate buffer, pH 7.4, containing 0.02% sodium azide to prevent bacterial growth (the caps of tested tubes were wiped with a swab soaked in toluene). A blank was prepared using only BSA in the same buffer. The reaction mixtures were incubated at 37 °C for twenty five days and were afterwards assayed to determine the presence of advanced glycation end products, such as fructosamine. Method of dialysis are given in the Supplementary Materials.

#### 2.6.2. Advanced Glycation End Products Formation

The fluorescence intensity of the reaction products was determined using the spectrofluorometric detector POLAR star Omega and a plate reader (BMG Labtech, Ortenberg, Germany), with excitation and emission wavelengths being 340 nm and 440 nm, respectively. The results are presented as fluorescence intensity and expressed as advanced glycation end products (AGEs) units: 1 U = fluorescence of 0.5 M glucose solution [23].

The natural luminescence of caramel has been previously noted in the literature [4]. We noted that the fluorescence of caramel was green in color, whereas the fluorescence of CQDs was blue, which also agrees with the previously described results [33]. We suggest that the caramel also contains CQDs; however, with different sizes and functional groups at the surface. It is known that these two parameters change the optical properties of CQDs [34,35]. Therefore, the measurements of biochemical interactions with the use of the photoluminescence method in those cases are not applicable.

#### 2.6.3. Fructosamine Content

The concentration of the Amadori product fructosamine was determined using an NBT assay [36] as detailed previously [37]. In brief, 30 μL of glycated BSA sample was mixed with 180 μL of 0.5 mM NBT in 100 mM of carbonate buffer, pH 10.4, and incubated in the dark at 37 °C for 15 min. The absorbance was measured at λ = 530 nm using a plate reader (ThermoFisher Scientific, Waltham, MA, USA). The fructosamine concentration was calculated from a 1-DMF standard curve (1–100 mmol/L).

### 2.7. Oxidative Stress in Adipose Tissue

#### 2.7.1. Isolation of Adipose Tissue and Incubation with Tested Solutions

Rat adipose tissue from around the kidney was collected as previously described by Erukainure et al., 2022, with minor modifications [25]. The freshly isolated tissue collected was immediately rinsed with Kreb’s buffer (125 mM NaCl, 5 mM KCl, 2.5 mM CaCl_2_, 1.25 mM KH_2_PO_4_, 2.5 mM MgCl × 6 H_2_O, 25 mM NaHCO_3_, 1 mM glucose) and cut into small pieces with each weighing 0.1 g. Each piece of adipose tissue was incubated with 2 mL of Kreb’s buffer containing the tested solutions, or with Kreb’s buffer (control). The incubation period was 2 h at 37 °C. The adipose tissue samples were collected from the buffer and homogenized in 1 mL cold 100 mM phosphate buffer containing 1% triton X-100 (pH 7.5) and centrifuged at 15,000 rpm for 10 min at 4 °C. The supernatants were decanted into 2 mL Eppendorf tubes and stored at −20 °C for subsequent biochemical analyses.

#### 2.7.2. Lipid Peroxidation Assay

The malondialdehyde (MDA) content was determined using a previously published method [38]. The level of thiobarbituric acid reactive substances (TBARS), a measure of lipid peroxidation, was determined using the TBA spectrophotometric assay [39]. Briefly, 250 μL of the tested supernatants, 500 μL of 15% TCA and 500 μL of 0.37% TBA, were mixed. The samples were incubated at 100 °C for 10 min and cooled and centrifuged at 10,000× *g* for 10 min. The absorbance was measured at λ = 535 nm. The MDA content was determined using a standard curve for TEP (1–5 nM) and was expressed in nmoles of TBA per mL.

#### 2.7.3. Total Reducing Power of Adipose Tissue

The assay was performed according to Benzie and Strain, 1999 [40], with some modifications [41]. The effect of the tested solutions on the total reducing power of adipose tissue was determined by spectrophotometric analysis of the concentration of reduced iron. In the experiment, 20 µL of the tested supernatants and 180 µL of the reagent were added to a solution consisting of 10 parts of a 0.3 M sodium acetate buffer, pH 3.7, 1 part of 0.01 M TPTZ solution and 1 part of 0.02 M FeCl_3_ × 6 H_2_O solution. After 10 min of incubation at room temperature, absorbance was measured at λ = 593 nm. The results have been presented as the amount of reduced iron ions (Fe(II)). FeSO_4_ × 7 H_2_O was used for the construction/creation of a standard curve (0.1 to 1 mM).

### 2.8. Ferric Reducing Antioxidant Power (FRAP)

The in vitro reducing activity of the tested solutions was determined using a similar method to that in Section 2.7.3. For this purpose, 20 µL of the tested solutions in PBS and 180 µL of the reagent were incubated [42,43].

### 2.9. In Vitro Integration of the Red Blood Cell Membrane

#### 2.9.1. Preparation of Blood Sample for Red Blood Cells Lysis Assay

Red blood cells were prepared as described by Parvin et al., 2015 [44], with minor modifications. Fresh whole blood was collected from rats into a glass tube containing 3.2% sodium citrate (Equimed, Krakow, Poland). All the blood samples were then mixed with an equal volume of Alsever solution (2% glucose, 0.8% sodium citrate, 0.5% citric acid and 0.42% NaCl) and stored at 4 °C for 24 h before use. The samples were centrifuged at 2500 rpm for 5 min and the supernatant was removed. The cell pellet was washed with 0.9% NaCl solution and centrifuged at 2500 rpm for 5 min. This was repeated three times until the supernatant was clear and colorless. The cellular component was reconstitued to a 20% suspension (*v*/*v*) with 100 mM sodium phosphate buffer, pH 7.4.

#### 2.9.2. Red Blood Cells Lysis Assay

The effects of the tested solutions on red blood cell lysis were measured as described by Li et al., 2015 [45], with minor modifications. Briefly, 200 μL of the red blood cells suspension was mixed with 20 μL of the tested solutions, or 100 mM sodium phosphate buffer, pH 7.4 (negative control), or 20 μL of water to yield complete hemolysis (positive control). After incubation for 20 h at 37 °C, the suspensions were centrifuged at 3000 rpm for 20 min, and the supernatants were collected. The absorbance of the released hemoglobin (sample size—200 μL) was measured at λ = 540 nm using a plate reader (ThermoFisher Scientific, Waltham, MA, USA). The presence of hemolysis was calculated as the percentage of hemolysis in the positive control (100%).

### 2.10. Statistical Analysis

All analysis was carried out in triplicates in two or three independent experiments and expressed as mean ± standard deviation (SD). Post hoc analysis using Dunnett’s multiple comparisons was performed using Graph Pad Prism Software (San Diego, CA, USA, version 8). Significance was set at *p* < 0.05.

## 3. Results

### 3.1. Carbon Quantum Dots and Caramel

**Figure 1:**
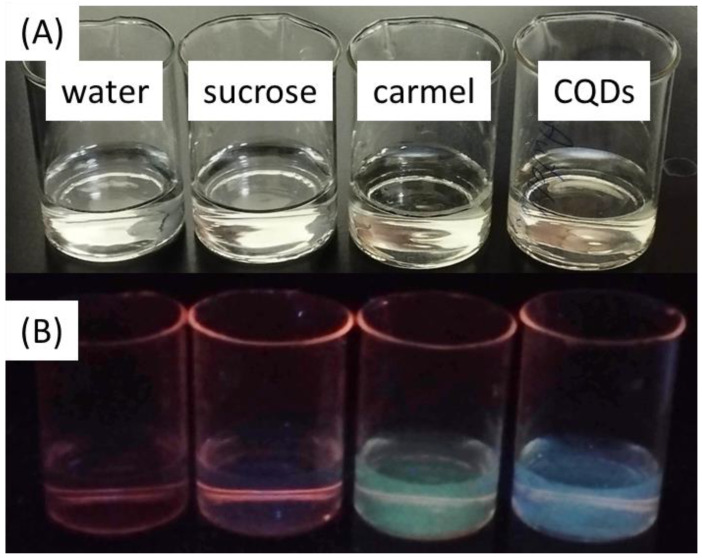
The solutions under (**A**) sunlight and (**B**) UV light.

Photographs of the solutions under sunlight are presented in Figure 1A. Both the caramel and CQDs were diluted 100 times. All these solutions were colorless. Under UV light (Figure 1B), both caramel (green) and carbon quantum dots (blue) are fluorescent. We suggest that due to incomplete thermal decomposition, caramel contains certain amounts of carbon quantum dots.

**Figure 2:**
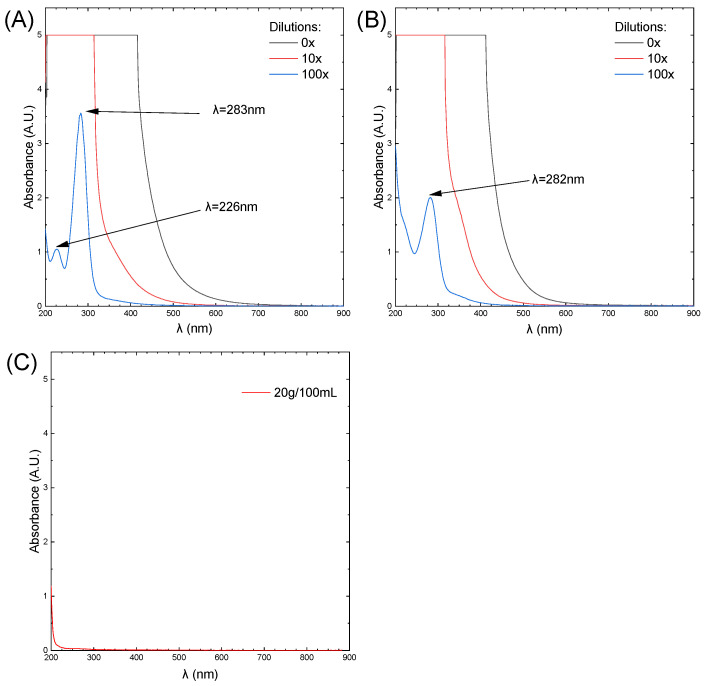
The UV-Vis spectra of: (**A**) caramel, (**B**) CQDs, (**C**) sucrose solutions.

The UV-Vis spectra of the caramel, CQDs, and sucrose solutions are shown in Figure 2. The spectra were recorded using a standard cuvette with an optical path length of 1 cm, with deionized water as a reference.

The spectra of caramel and CQDs are similar. Both have a distinct peak at a wavelength of 283 and 282 nm, respectively. In the case of caramel, there is also a low-intensity peak at a wavelength of 226 nm, which is not present in the CQD spectrum. However, in the visible range, there is a bend. This suggests that due to the further thermal decomposition of sucrose, new structures are formed. In this case, we consider them to be carbon quantum dots.

The presence of a distinct absorption band in CQDs and caramel samples makes it difficult to read absorbance during some bio-chemical test (cytotoxicity). Therefore, the UV-Vis spectra presented were used to determine the extinction coefficients for the various wavelengths used in bio-chemical measurements. Based on the assumption that absorbance is an additive quantity according to Lambert–Beer’s law, the component of absorbance from CQDs and caramel was subtracted from the results, taking into account their concentration in each sample.

### 3.2. Effect of Carbon Quantum Dots and Caramel on Platelet Aggregation

**Figure 3:**
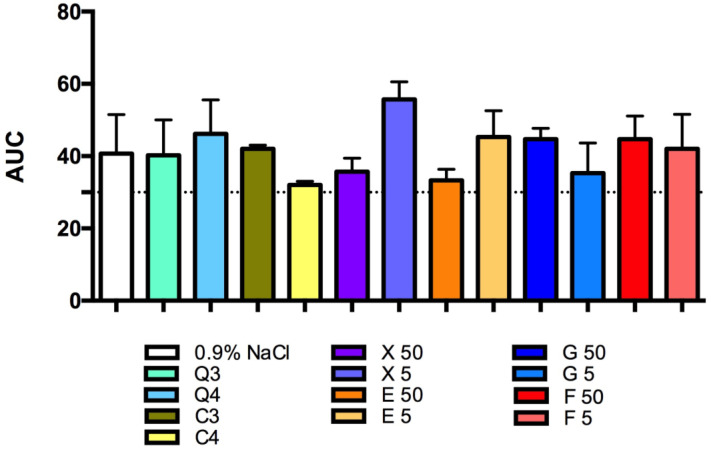
Effects of the tested solutions of CQDs, caramel, glucose, fructose, erythritol and xylitol on in vitro whole rat blood aggregation induced by collagen (1.6 µg/mL). The results are expressed as mean ± SD, *n* = 3–10, one-way ANOVA, post hoc Dunnet test. AUC—area under curve.

The CQDs and caramel, each diluted by 100× and 30×, did not influence platelet function when added to a mixture of whole blood/saline and incubated for 3 min (Figure 3). Higher concentrations were not tested as it is impossible to obtain such high concentrations from the basal solution using this method.

The glucose, fructose, erythritol and xylitol solutions tested did not influence platelet aggregation (Figure 3).

The figure containing the results for reference compounds that inhibit and intensify aggregation in comparison to CQDs and caramel is given in the Supplementary Materials (Figure S1).

### 3.3. Effect of Carbon Quantum Dots and Caramel on Platelet Viability

**Figure 4:**
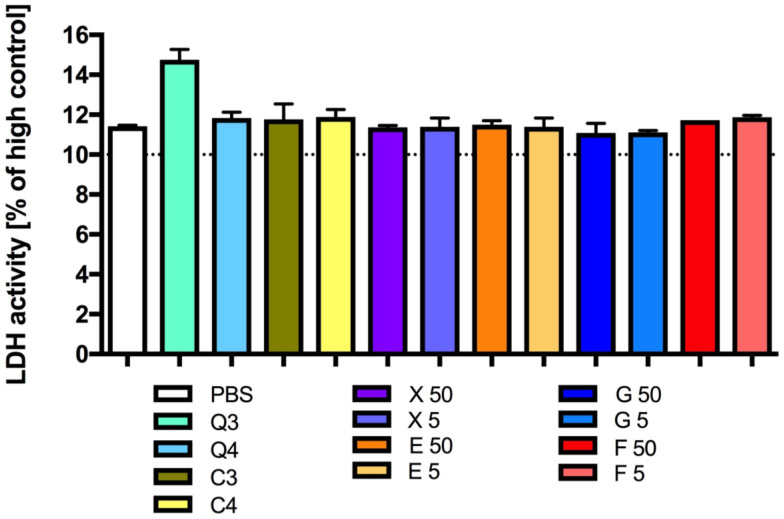
Platelet viability: LDH activity after incubation of platelets with CQDs, caramel, glucose, fructose, erythritol or xylitol solutions. Mean +/− standard deviation (SD), *n* = 3, Kruskal–Wallis test (ANOVA), and Dunn’s post hoc test. PSB—0.1 M sodium phosphate buffer; high control—lysis (medium + lysis solution).

The lactate dehydrogenase activity in platelet-rich plasma incubated with the CQD solutions tested was determined to be comparable to the lactate dehydrogenase activity of the control sample (Figure 4). In the samples incubated with different concentrations of caramel or with the sucrose solution, there was also no difference in lactate dehydrogenase activity compared to the control sample.

The lactate dehydrogenase activity in platelet-rich plasma incubated with the tested glucose, fructose, erythritol and xylitol solutions was determined to be at a similar level to the lactate dehydrogenase activity in platelet-rich plasma incubated with the medium alone, namely, the control sample (Figure 4).

### 3.4. Effect of Carbon Quantum Dots and Caramel on Protein Glycation

**Figure 5:**
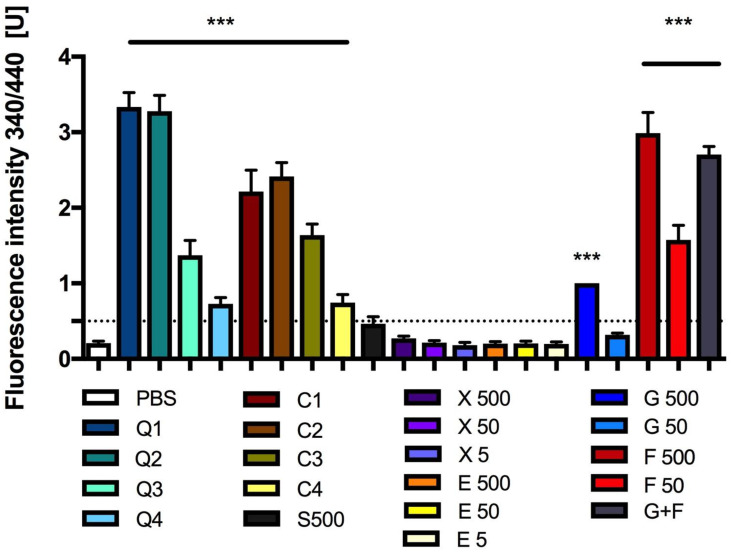
Fluorescence (340/440) after incubation of BSA with CQDs, caramel or sucrose, glucose, fructose, erythritol or xylitol solutions. Mean +/− standard deviation (SD), *n* = 4, one-way ANOVA, and Dunnett’s post hoc test, *** *p* < 0.001. PSB—0.1 M sodium phosphate buffer. 1 U = fluorescence 500 mM glucose solution.

Firstly, the complete formation of AGEs after 25 days of incubation of the tested solutions with BSA was determined by measuring the fluorescence intensity (Figure 5).

The incubation of 500 mM or 50 mM glucose with BSA increased the fluorescence intensity by approximately 377% and 52%, respectively, when compared to BSA + PBS fluorescence. The fluorescence intensity of BSA incubated with 500 mM or 50 mM fructose increased significantly by about 1325% and 660%, respectively, when compared to BSA + PBS fluorescence. After the incubation of BSA with 250 mM glucose + 250 mM fructose, the fluorescence was increased by about 1190% when compared to BSA fluorescence (Figure 5).

The fluorescence intensity values after xylitol (50 or 5 mM) and erythritol (500, 50 or 5 mM) incubation with BSA were similar to those of BSA + PBS fluorescence (Figure 5).

The results obtained after measuring the fluorescence intensity in samples containing various concentrations of caramel and CQDs are presented in Figure 5. However, we did not take into account that these are glycation results, and therefore we present the data as only a confirmation of caramel and CQDs’ fluoresce under these conditions. In the supplement, we have also given the results from an experiment in which we tried to separate glycated BSA from CQDs or caramel, so that only glycated BSA would remain in the system, and to count the signal after glycation by CQDs or caramel. Unfortunately, the CQDs or caramel did not pass through the dialysis membrane, which indicates that they can combine with BSA (Figure S2).

**Figure 6:**
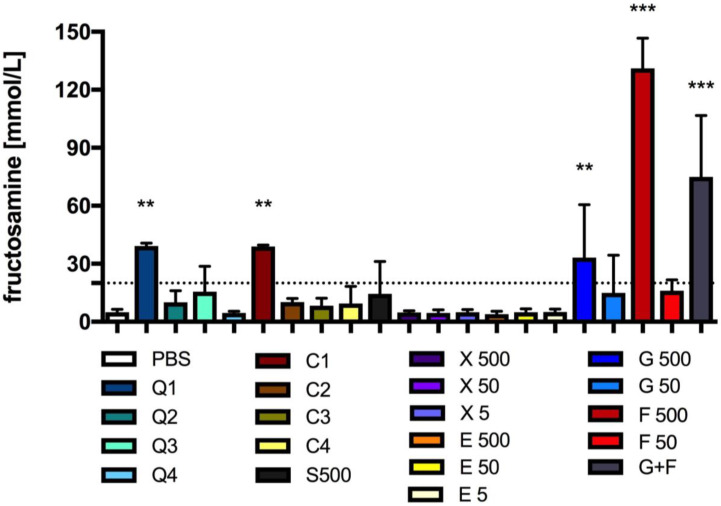
Fructosamine levels after incubation of BSA with CQDs, caramel or sucrose, glucose, fructose, erythritol or xylitol solutions. Mean +/− standard deviation (SD), *n* = 4, one-way ANOVA, and Dunnett’s post hoc test, ** *p* < 0.01, *** *p* < 0.001. PSB—0.1 M sodium phosphate buffer.

After day 25 of incubation, the fructosamine levels in BSA incubated with CQDs or caramel (10×, 30×, 100× diluted) were at approximately the same level as in the sample with only BSA + PBS (Figure 6). The level of these glycated proteins was the highest in the samples with the most concentrated CQDs or caramel solution, but much lower (over three times) than in the sample in which 500 mM fructose was incubated. The levels of fructosamine in the BSA samples with 500 mM fructose (F 500), 250 mM glucose and 250 fructose (G + F), and 500 mM glucose (G 500) were about 2500%, 1400% and 570% higher, respectively, than in the control BSA + PBS sample (Figure 6). In the other groups, no differences in fructosamine levels were established when compared to the control group (Figure 6).

### 3.5. Effect of Carbon Quantum Dots and Caramel on Lipid Peroxidation

**Figure 7:**
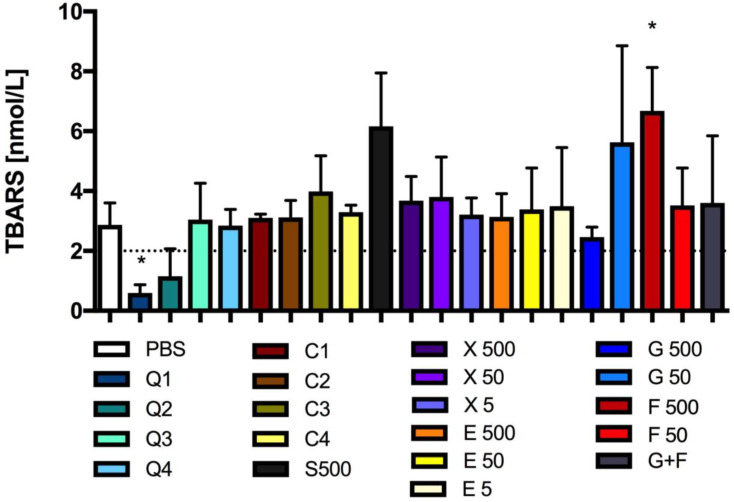
Lipid peroxidation: TBARS concentration after incubation of adipose tissue with CQDs, caramel or sucrose, glucose, fructose, erythritol or xylitol solutions. Mean +/− standard deviation (SD), *n* = 3, one-way ANOVA, and Dunnett’s post hoc test, * *p* < 0.05. PSB—0.1 M sodium phosphate buffer.

The CQD solution with a concentration three times lower than the concentration of the basal solution significantly decreased lipid peroxidation in adipose tissue. The thiobarbituric acid reactive substance (TBARS) concentration was significantly lower in this sample in comparison to the concentration in the control sample (Figure 7). In the sample where the adipose tissue was exposed to a CQD concentration approximately three times lower, the level of TBARS was approximately 40% of the TBARS level determined in the control sample.

The incubation of adipose tissue with a 500 mM fructose solution resulted in a significant increase in the TBARS level by approximately 200% of the level determined in the control sample. The other tested solutions showed no significant activity in the MDA test (Figure 7).

**Figure 8:**
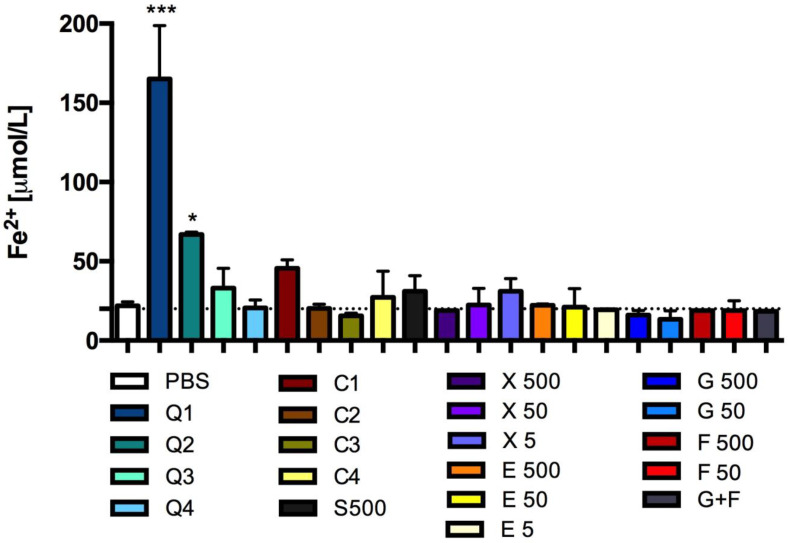
Reducing activity: Fe^2+^ concentration after incubation of adipose tissue with CQDs, caramel or sucrose, glucose, fructose, erythritol or xylitol solutions. Mean +/− standard deviation (SD), *n* = 3, one-way ANOVA, and Dunnett’s post hoc test, * *p* < 0.05, *** *p* < 0.001. PSB—0.1 M sodium phosphate buffer.

CQDs also showed reducing activity. After the incubation of CQD solutions (3- and 10-times diluted basal solution) with adipose tissue, the Fe(II) levels significantly increased (Figure 8). The high concentration of caramel also caused an increase in the formation of these ions in the tested system, which was approximately 100% more than in the control sample. The other solutions tested did not affect the reducing activity of adipose tissue (Figure 8).

### 3.6. In Vitro Reducing Properties of Carbon Quantum Dots and Caramel

**Figure 9:**
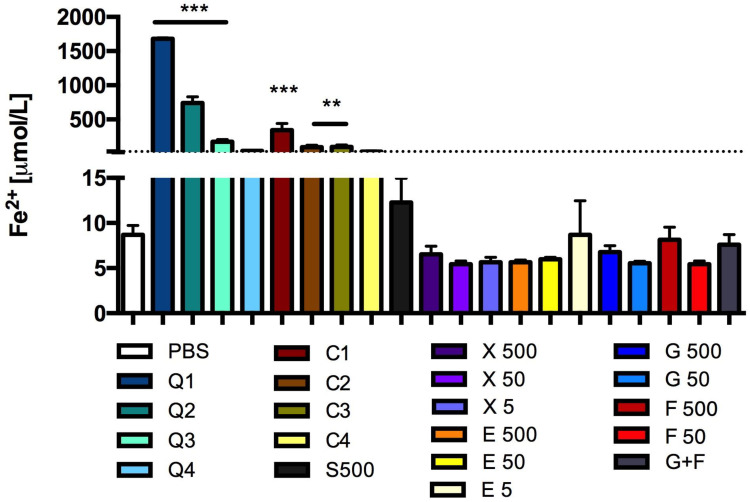
In vitro reducing activity: Fe^2+^ concentration after incubation of iron (III) solution with CQDs, caramel or sucrose, glucose, fructose, erythritol or xylitol solutions. Mean +/− standard deviation (SD), *n* = 4, one-way ANOVA, and Dunnett’s post hoc test, ** *p* < 0.01, *** *p* < 0.001. PSB—0.1 M sodium phosphate buffer.

In the in vitro FRAP test, the three most concentrated solutions of CQDs caused a concentration-dependent reduction in iron (III) ions. Similar results were obtained for caramel, but its activity was about three times weaker (Figure 9).

The other sugar and sweetener solutions tested did not affect the amount of Fe (II) ions produced. The results obtained (Figure 9) were comparable to those of the control solution (PBS).

### 3.7. Effect of Carbon Quantum Dots and Caramel on Integrity of the Red Blood Cell Membrane

**Figure 10:**
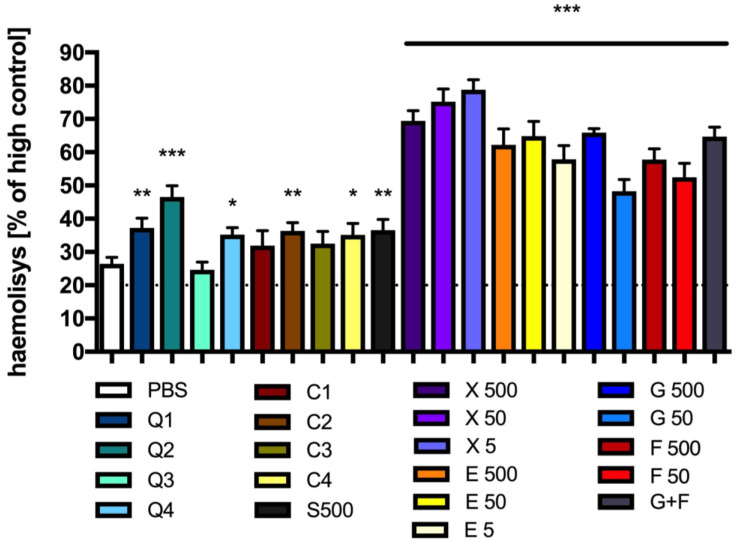
Red blood cell haemolysis: Absorbance of sample after incubation of red blood cells with CQDs, caramel or sucrose, glucose, fructose, erythritol or xylitol solutions. Mean +/− standard deviation (SD), *n* = 3, one-way ANOVA, and Dunnett’s post hoc test, * *p* < 0.05, ** *p* < 0.01, *** *p* < 0.001. PSB—0.1 M sodium phosphate buffer, Hight control—100% hemolysis (red blood cells + water).

The incubation of red blood cells with PBS for twenty hours led to approximately 25% hemolysis when compared to the hemolysis observed in the control sample, where the blood cells were incubated with water (100% hemolysis). The hemolysis of red blood cells after incubation with CQDs or caramel solutions did not exceed 40% of the maximum hemolysis. An exception is the incubation with CQDs of a tenfold dilution of the starting solution, where the hemolysis was lower than 50% of the maximal hemolysis (Figure 10).

The incubation of red blood cells with solutions of glucose and/or fructose resulted in an approximately 50% or more increase in hemolysis (Figure 10). The incubation of xylitol or erythritol solutions with red blood cells for 20 h caused hemolysis of blood cells in the range of 50–75% of the maximum (Figure 10).

## 4. Discussion

Caramel, which is both a coloring agent and an antioxidant, is used in several food products and is consumed by many people in varying amounts [4]. Our research shows that the caramelization of sucrose under special conditions leads to the formation of CQDs. As a result, humans also consume CQDs. Therefore, it is theoretically possible for these particles to affect the body. Despite an increasing number of studies describing different types of carbon quantum dots, their biosafety is still not clearly understood. In our in vitro research, we examined the effects on platelet aggregation, protein glycation and lipid peroxidation of CQDs and caramel formed from a 20% (mass/volume) sucrose solution.

Subsequently, if patients with metabolic diseases consume excess sugars and their metabolites, disturbances in platelet aggregation, the excessive glycation of proteins and the breakdown of antioxidant defence mechanisms occur, leading to, for example, lipid peroxidation, which contributes to the severity of the disease [46]. Metabolic diseases are a global medical and economic problem, meaning that the number of patients exposed to the adverse effects of sugar metabolites is constantly increasing.

We also tested, under the same reaction conditions, the impacts of different concentrations of glucose, fructose, erythritol and xylitol, as well as sucrose (although it is known that it is not fully absorbed and cannot act on its own in vivo, out of curiosity, we decided to investigate its effect in vitro), on some of these processes.

It is known that hyperglycemia is associated with platelet activation. It has been shown that platelets from chronically hyperglycemic patients present higher expressions of adhesion molecules on their surfaces and enhanced thromboxane production, and are hyperreactive [47,48]. High glucose levels enhance platelet reactivity via multiple mechanisms. Chronically elevated blood glucose promotes non-enzymatic protein glycation, forming glycated or oxidized products, which alter the oxidation–reduction potential and elicit platelet hyperactivation [47,49].

The results of previously published studies show that glucose, fructose and, more recently, erythritol may enhance platelet reactivity in a concentration-dependent manner [2,47,50]. Additionally, erythritol can be produced endogenously from glucose [51], which may lead to a stronger effect than expected by the patient on, for example, blood platelets, becoming dangerous in patients with metabolic disorders.

The literature contains several descriptions of studies on the antiplatelet and antithrombotic effects of CQDs. Depending on the starting material used to generate CQDs, they can shorten bleeding time, increase the number of platelets, activate the coagulation system, inhibit platelet activation, etc. [52,53,54,55]. In our research, in order to evaluate the influence of the tested substances on platelet aggregation (after induction with collagen), freshly isolated rat whole blood was incubated with the tested substances or vehicle, and the aggregation responses were assessed with a whole blood aggregometer by measuring impedance change. The CQDs or caramel tested using this method did not affect platelet activity at all the doses tested. Importantly, CQDs and caramel did not affect the survival of platelets, similarly to the other tested sugars and sweeteners.

Surprisingly, we did not show any marked effects of high glucose (and other sweeteners) concentrations on platelet aggregation. It is known that one of the mechanisms by which hyperglycemia elicits thrombogenic effects is associated with non-enzymatic protein glycation and oxidation; however, these processes may not occur in short-term in vitro aggregation studies. On the other hand, there are also studies showing that acute hyperglycaemia may also influence platelet function. Studies by Sudic et al. [47] showed that high glucose levels after 20 min of incubation enhanced ADP and TRAP platelet activation in whole blood. Studies by Tang et al. showed that high glucose concentrations after 90 min of incubation increased the aggregation induced by collagen, but not ADP, in PRP [48]. However, studies by Massucco et al. [56] showed that a high glucose concentration did not alter the response to collagen and ADP after 6 min of incubation. Therefore, the discrepancies in results may be an effect of the different experimental conditions, the differences in the concentrations of aggregation inducers when using PRP versus whole blood, or the differences in the incubation time. It has been shown that acute hyperglycemia increases platelet membrane-bound protein kinase C (PKC) activity, probably because of osmotic stress [56,57]. A detailed study performed by Massucco et al. showed that short-time incubation with a high glucose concentration (as well as iso-osmolar mannitol) did not modify the platelet response and aggregation to collagen and ADP. This was probably due to the fact that high glucose concentration (as well as iso-osmolar mannitol or fructose) causes the translocation of beta-isoforms of PKC to the platelet membrane with an osmotic mechanism [56,57]. There is evidence that PKC activation increases calcium concentration in platelets and intracellular calcium activates eNOS, which results in the stimulation of the NO/cGMP pathway in platelets [56]. This effect may be responsible for the lack of pro-aggregating effects of high concentrations of glucose and other sweeteners observed in our study. Of note, the role of the osmotic effect on PKC activation strictly depends on experimental conditions. In the studies performed by Massuco et al., the maximal increase in NO production in platelets incubated with high concentrations of glucose (or iso-osmolar mannitol) was transient, with the maximum observed between 6 and 20 min, and these disappeared after 60 min of incubation [56]. In our study, the incubation time was 3 min, and the transient production of NO may be responsible for the lack of enhancement of platelet response to collagen observed in our study. It should also be noted that the experiments were performed in rat whole blood, where other blood cells in addition to platelets are present and may modulate platelet function. Therefore, this is a limitation of our study, and further studies are necessary to fully explain the potential interactions between CQDs from caramel and platelets, including studies on human platelets, with different incubation periods and different agonists and at different concentrations.

Glycation and peroxidation are known to play a key role in the complications of many pathophysiological processes. Glycation is a non-enzymatic reaction between the carbonyl group of reducing sugars and a free amino group of proteins, resulting in the formation of AGEs [58] and of the reactive intermediate products of glycoxidation (ROS and a-dicarbonyls) [59]. Chronically elevated blood glucose levels may facilitate the creation of AGEs [60]. AGEs can be formed via multiple pathways from reducing sugars, e.g., the auto-oxidation of reducing sugars, a reaction initiated by the Schiff base and a reaction proceeding from a ketoamine or Amadori product [61,62]. The final irreversible forms of AGEs are either fluorescent cross-linked structures or non-fluorescent structures [61,62,63]. The elevated concentrations of glucose and reactive a-dicarbonyl groups may activate the polyol pathway endogenously, and bring about the conversion of glucose to sorbitol and then to fructose, thus further contributing to the formation of AGEs [61,64]. These glycation products can act on inflammatory and/or vascular cells (e.g., by the release of pro-inflammatory mediators) and cause cardiovascular dysfunctions [65,66], such as damage to myocardial tissue or fibrosis.

Interestingly, foods processed at high temperatures by grilling, frying, or baking undergo the Maillard reaction, which enhances flavor, taste, and appearance, but which also forms AGEs [67]. The absorption of exogenous AGEs remains unclear; however, metabolic studies have shown that approximately 10–30% of the total exogenous AGEs consumed are absorbed by the intestine, of which two-thirds accumulate in tissue [68,69].

Our research shows that CQDs and caramel at high concentrations can induce the formation of AGEs, as shown by the measurement of the level of fructosamine in samples incubated with BSA. However, this is not as intense as the induction of fructosamine formation when using fructose or glucose + fructose, where the concentration of marked fructosamine was more than three times higher. Our research shows that fluorescent AGEs were also formed when BSA was incubated with glucose and xylitol (at very high concentrations). This shows that although fructosamine levels reflect the amount of AGEs in blood, their measurement does not provide a comprehensive picture of the severity of glycation. Therefore, the extent of the effect of CQDs from caramel or caramel itself on protein glycation remains to be extensively investigated. Unfortunately, our study indicates that the formation of AGEs during CQDs’ or caramel’s action on protein is possible.

In the hyperglycemic state, glucose can be metabolized to compounds that alter the oxidation–reduction potential and intracellular signaling pathways [70,71]. Redox disorders in the course of metabolic diseases have serious consequences, namely, the intensification of existing disorders, the development of complications such as chronic renal failure and atherosclerosis [72,73], and serious vascular complications. Possible causes of oxidative stress and damage to proteins in hyperglycemia include free radicals, generated by autoxidation reactions of sugars and sugar adducts to protein, and the autoxidation of unsaturated lipids in plasma and membranes. Oxidative stress may be amplified by a continuing cycle of metabolic stress, leading to increased free radical production, and compromised free radical inhibitory and scavenger systems [70].

Several previous studies have shown that CQDs can have reducing and antioxidant activities [41,74,75,76], two properties that are of great importance. Our research also showed that the CQDs synthesized from caramel can reduce lipid peroxidation and have a reducing effect in adipose tissue, as well as in vitro. It is interesting to note that in our study, fructose significantly induced the formation of TBARS during incubation with adipose tissue. Glucose and sucrose also slightly increased the formation of TBARS, whereas caramel, erythritol and xylitol did not exert any significant effects. Since CQDs also have antioxidant properties, unlike the other tested sugars (also at the concentration at which they cause protein glycation in vitro), their intake appears to be safer than the intake of sugars such as fructose or glucose. However, extensive research is required to establish the direct interactions between the effects of glycation and oxidation after the ingestion of CQDs. Interestingly, caramel had the strongest effects of the three concentrations tested, also showed a reducing effect in vitro. This shows a significant difference between the reducing power of caramel or caramel CQDs and the sweeteners and sugars tested.

It was assumed that blood contact biomaterials such as carbon quantum dots will inevitably interact with red blood cells (RBCs), as shown by previous research results [45]. After all, it should be remembered that red blood cells, CQDs and caramel are all loaded with a negative surface charge [77]. Such interactions show repulsion, so potential interactions will be weak or unobservable. Moreover, because of their high abundance in blood, RBCs are a good model for preliminary investigations into the complicated interactions between mammalian cell membranes and foreign biomaterials [45]. Hemolysis refers to the release of hemoglobin from RBCs. The disturbance of the membrane integrity of RBCs and influence on hemolysis has been widely used in biosafety evaluations of various biomedical materials [45].

Our study shows that the incubation of the tested sugars, sweeteners, CQDs or caramel with red blood cells for 20 h may influence RBC membrane integrity. Hemolysis after incubation with CQDs or caramel was the least severe, with up to twice the amount of hemolysis observed in the control sample (incubated with PBS). It is known that during routine storage, biochemical and biomechanical changes occur in RBCs. These changes, collectively referred to as the RBC storage lesion, include increased hemolysis, reversible and irreversible RBC shape changes, membrane vesiculation, altered aggregability and decreased deformability [78]. The loss of erythrocyte antioxidant activity during RBC storage has been demonstrated previously, and it is plausible that the addition of antioxidants to stored RBCs has the potential to mitigate oxidative injury [78]. Our research shows that CQDs and caramel have a significant reducing effect in vitro (FRAP test); therefore, for this reason it is possible that the hemolysis observed after their incubation with blood cells was less severe than the hemolysis observed in the other samples containing sugars or sweeteners.

For our in vitro studies, based on the literature, we selected high concentrations of these sugars, such as 500 mM or 50 mM, as they can significantly disturb aggregation, glycate proteins, and intensify lipid peroxidation at these concentrations [23,24,25,26,27,28]. A level of hyperglycemia of about 50 mM is not clinically irrelevant, as blood glucose levels are commonly around 30 mM in diabetic ketoacidosis, and can reach approximately 60 mM during diabetic hyperosmolar coma [79]. Such high concentrations (about 50 mM) of sugars and sweeteners other than glucose are unlikely to be reached in the body; however, this depends on the amount consumed. Therefore, we would like to emphasize that these are initial tests determining whether it is possible for CQDs and caramel to influence the selected processes.

In conclusion, our study shows that caramel and the CQDs formed from caramel may influence the glycation process and integrity of the red blood cell membrane, but unlike glucose and fructose, they decrease lipid peroxidation and may reduce Fe (III), thus having antioxidant properties. Additionally, it is unlikely that they affect platelet aggregation. Compared to glucose and fructose, they may be safer for patients with metabolic disorders; however, further research is needed on the safety and biological activity of caramel and CQD.

## Data Availability

Data supporting the reported results are available from the corresponding authors upon written request.

## Supplementary Materials

The following supporting information can be downloaded at: https://www.mdpi.com/article/10.3390/antiox13010013/s1, Figure S1: Effects of the tested solutions of CQDs (Q3, Q4), caramel (C3, C4), and reference compounds: guanabenz and aspirin (ASA) on in vitro whole rat blood aggregation induced by collagen (1.6 µg/mL). Results are expressed as mean ± SD, *n* = 3–10, one-way ANOVA, post hoc Dunnet test, * *p* < 0.05, ** *p* < 0.01, *** *p* < 0.001. AUC—area under curve; Figure S2: The fluorescence of the samples (BSA 10 mg/mL; fructose 500 mM; C1–C4, Q1–Q4), BSA+F500, BSA+C1, BSA+C2, BSA+C3, BSA+C4, BSA+Q1, BSA+Q2, BSA+Q3, BSA+Q4 after 5 days of incubation, and BSA+C4, BSA+Q1, BSA+Q2, BSA+Q3, BSA+Q4 after 5 days of incubation and after dialysis (two fractions).
