# Supplementary material for: The Influence of Caramel Carbon Quantum Dots and Caramel on Platelet Aggregation, Protein Glycation and Lipid Peroxidation
Source: Antioxidants (Basel). 2023 Dec 20;13(1):13. doi: 10.3390/antiox13010013 (PMC10812612; doi:10.3390/antiox13010013)
Supplement: Supplementary file 1 [file antioxidants-13-00013-s001.zip › antioxidants-2715361-supplementary.pdf]

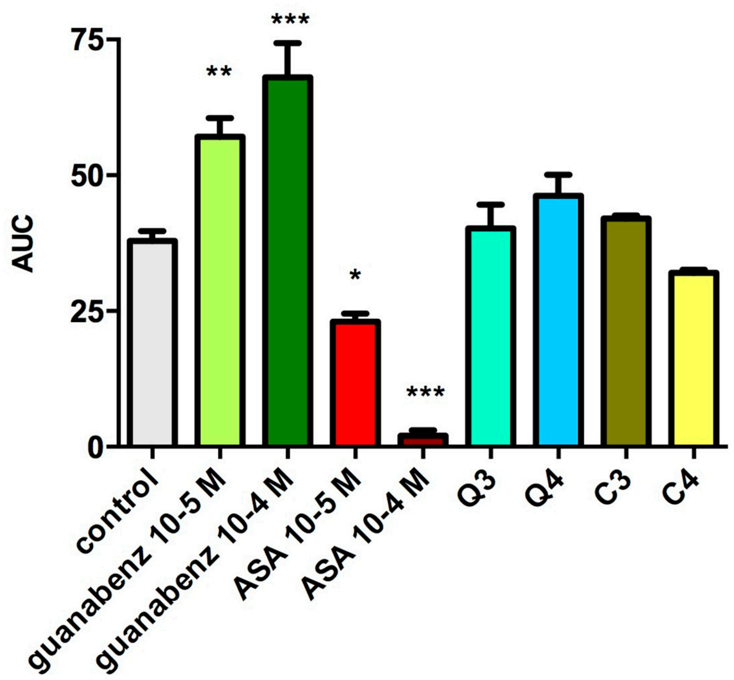

Figure S1. Effects of the tested solutions of CQDs (Q3, Q4), caramel (C3, C4), and reference compounds guanabenz and aspirin (ASA) on in vitro whole rat blood aggregation induced by collagen (1.6 μg/mL). Results are expressed as mean ± SD, n = 3-10, one-way ANOVA, post hoc Dunnet test, *p<0.05, **p<0.01, ***p<0.001. AUC—area under curve.

S1. Measerement of fluorescene intensity of some samples, samples alfer incubation with BSA, samples after incubation with BSA and dialysis.

S1.1. Methods

S1.1.1. Preparation of glycated BSA samples

In brief, BSA (10 mg/mL in 100 mM sodium phosphate buffer, pH 7.4) was incubated with the tested solutions in 100 mM sodium phosphate buffer, pH 7.4, containing 0.02% sodium azide to prevent bacterial growth (caps of tested tubes were wiped with a swab soaked in toluene). A blank was prepared using only BSA in the same buffer. The reaction mixtures were incubated at 37 °C for five days and were afterwards assayed to determine the presence of advanced glycation end products and fructosamine.

S1.1.2. Dialysis of solutions after the glycation process.

After the incubation process, the reaction mixtures underwent dialysis utilizing a membrane with a permeability range of 12-14,000 daltons (Medicell International Ltd, Liverpool, London, UK). The dialysis membrane, featuring a 28.6 mm-diameter tube, was trimmed to the requisite length and sealed on one end with a dedicated clip. Subsequently, the reaction mixture containing tested solutions after the incubation process was meticulously introduced into the inside of the vessel thus created, and the opposing end was sealed with another clip. The resulting system was immersed in a beaker filled with distilled water on a magnetic stirrer. The side from which the solution was introduced was affixed to the beaker wall above the liquid level using a clip. Stirring occurred at a rate of 300 RPM. Dialysis transpired over 24 hours, during which the water in the beaker was changed three times. The temperature during the process was 20 °C. The dialysis solution had a volume of 10 cm3, while the beaker water volume remained at 1 dm3. This procedure was replicated for each incubated reaction mixture. The fluorescence intensity of the reaction products was determined using a spectrofluorometric detector POLAR star Omega and a plate reader (BMG Labtech, Ortenberg, Germany), with excitation and emission wavelengths of 340 nm and 440 nm, respectively.

S1.2. Results

The results are shown in Figure S2.
1. CQDs or caramel fluoresce spontaneously under the tested conditions (excitation/emission wavelengths 340/440), proportional to the concentration (1);
2. Fructose does not fluoresce (weakly (1)), but after incubation with BSA the fluorescence increases significantly (2), indicating glycation of BSA by it;
3. After incubating CQDs or caramel with BSA, the fluorescence of the samples increases (2);
4. It is impossible to separate CQDs or caramel from BSA by dialysis, while in test samples (3) fluorescence is significantly higher than fluorescence in samples from the second fraction, water (4);
5. CQDs or caramel can cause glycation of BSA depending on the concentration, but we cannot determine its intensity by measuring fluorescence.

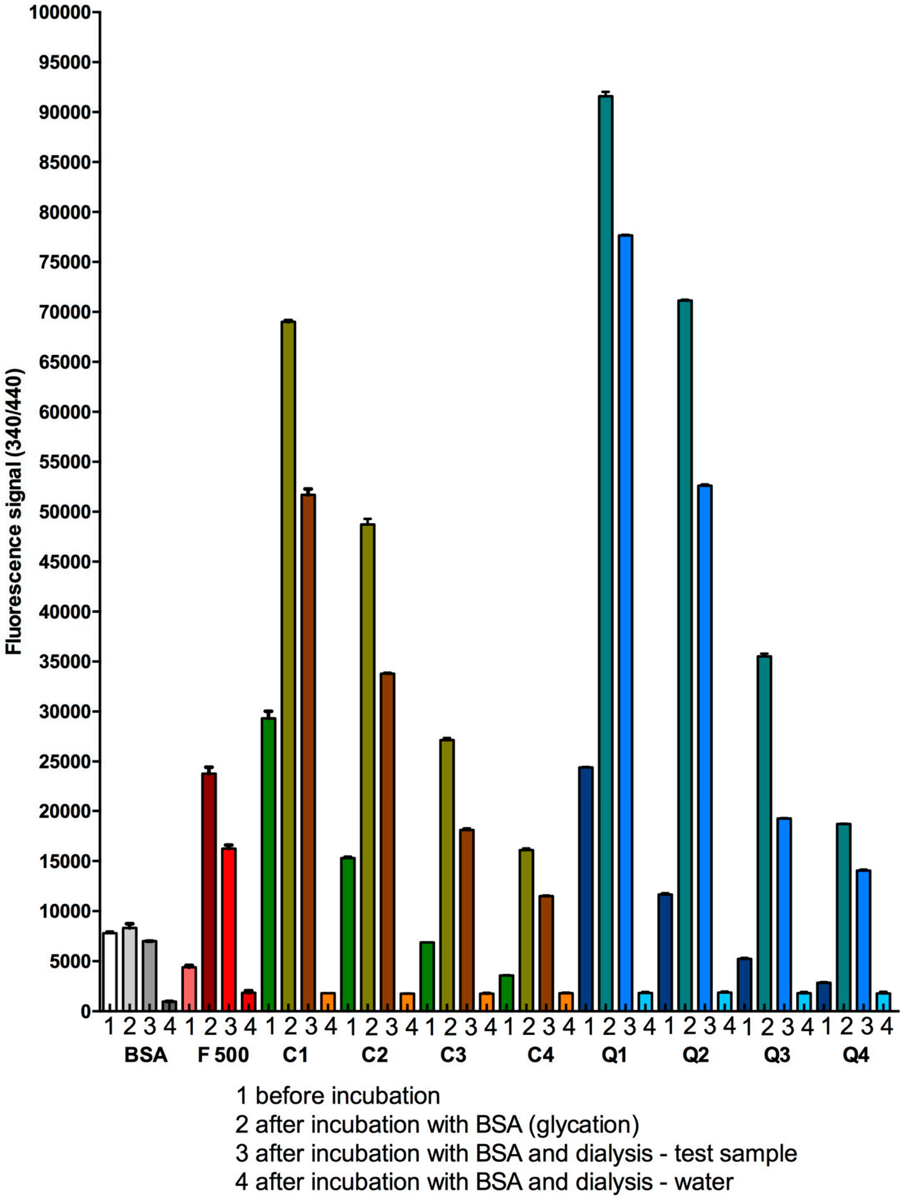

Figure S2. The fluorescence of the samples (BSA 10 mg/mL; fructose 500 mM; C1-C4, Q1-Q4), BSA+F500, BSA+C1, BSA+C2, BSA+C3, BSA+C4, BSA+Q1, BSA+Q2, BSA+Q3, BSA+Q4 after 5 days of incubation, and BSA+C4, BSA+Q1, BSA+Q2, BSA+Q3, BSA+Q4 after 5 days of incubation and after dialysis (two fractions).
